# Insecticide resistance and its underlying mechanisms in field populations of *Aedes aegypti* adults (Diptera: Culicidae) in Singapore

**DOI:** 10.1186/s13071-014-0471-0

**Published:** 2014-10-11

**Authors:** Sin-Ying Koou, Chee-Seng Chong, Indra Vythilingam, Chow-Yang Lee, Lee-Ching Ng

**Affiliations:** 1 Environmental Health Institute, National Environment Agency, 11 Biopolis Way, Helios Block, 138667 Singapore, Singapore; 2 Urban Entomology Laboratory, Vector Control Research Unit, School of Biological Sciences, Universiti Sains Malaysia, 11800 Penang, Malaysia; 3 Parasitology Department, Faculty of Medicine, University of Malaya, 50603 Kuala Lumpur, Malaysia

**Keywords:** Pyrethroid, Organophosphate, Synergist, Biochemical assay, Singapore, Dengue, *Aedes*

## Abstract

**Background:**

In Singapore, dose–response bioassays of *Aedes aegypti* (L.) adults have been conducted, but the mechanisms underlying resistance to insecticides remain unclear. In this study, we evaluated insecticide resistance and its underlying mechanism in field populations of *Ae. aegypti* adults.

**Methods:**

Seven populations of *Ae. aegypti* were collected from public residential areas and assays were conducted according to WHO guidelines to determine their susceptibility to several commonly used insecticides.

**Results:**

Various levels of pyrethroid resistance (RR_50_ = 3.76 to 142.06-fold) and low levels of pirimiphos-methyl resistance (RR_50_ = 1.01 to 1.51-fold) were detected. The insecticide susceptibility profile of *Ae. aegypti* adults was homogenous among the different study sites. Addition of the synergists piperonyl butoxide, *S,S,S*,-tributyl phosphorotrithioate, and triphenyl phosphate generally failed to enhance the toxicity of the insecticides investigated, suggesting an insignificant role of metabolic-based insecticide resistance and possible involvement of target site resistance. Further biochemical investigation of specific metabolic enzyme activities provided further evidence that detoxifying enzymes such as mono-oxygenases, esterases, glutathione S-transferases and altered acethylcholinesterases generally did not contribute to the resistance observed.

**Conclusions:**

This study confirmed the presence of pyrethroid resistance among *Ae. aegypti* adults in Singapore and documented the early onset of organophosphate resistance.

## Background

Dengue continues to be one of the most serious vector-borne diseases in the world. The global situation of dengue is well documented [1]. Thousands to hundreds of thousands of dengue cases occur every year in each of the many dengue-endemic countries, including Singapore, and the complex dengue epidemiology is a major challenge in managing the epidemics [2]. A proactive vector control approach is required to achieve effective and sustainable control of this disease [3], as there is still no vaccine or specific treatment for dengue.

Dengue viruses are transmitted by *Aedes* mosquitoes, and *Aedes aegypti* (L.) is the primary dengue vector in Singapore. This species has diurnal blood-feeding behaviour, with peak activities in the early morning and late afternoon. It is highly anthropophilic and displays a preference to feed and rest indoors or in close proximity to their breeding sites [4,5]. Thus, they are highly abundant in urban areas, propagating in and around human dwellings. Detection of the adult can be difficult, where they can rest undisturbed in sheltered areas.

Space spraying with adulticides is a common practice implemented in many dengue-endemic areas and this control method is necessary especially in areas with high mosquito density. The choice of space spraying techniques, such as ultra-low-volume application, thermal fogging, and indoor residual spraying, depends on the setting and field conditions to ensure maximum control. These treatments target the behaviour of *Ae. aegypti,* which usually rest on wall surfaces, thus increasing contact exposure of insecticides to achieve control [4]. However, the difficulty of achieving full coverage of the adult mosquito habitat limits the efficacy of application. Adults tend to rest hidden in sheltered locations, where insecticide may not reach. Furthermore, appropriate timing for fogging has often not been taken into account, although several models for effective fogging have been published in the last two decades [6,7].

The extensive and probably inappropriate application of insecticides has led to the development of insecticide resistance in *Ae. aegypti*. This species exhibits varying degrees of resistance to different insecticides. The numerous reports on the different types of resistance mechanisms have raised awareness on the importance of a good understanding of the resistance mechanisms for effective vector control.

Two important insecticide resistance mechanisms exhibited by insects are metabolic-based resistance and target site insensitivity [8-10]. The former involves three groups of detoxifying enzymes: mono-oxygenases (MFOs), esterases (ESTs), and glutathione S-transferases (GSTs) [11]. The target site insensitivity is associated with modification of three target sites: voltage-gated sodium channels, gamma-aminobutyric acid (GABA) receptors and acethylcholinesterases [12].

The objective of this study was to assess the extent of insecticide resistance and characterize the underlying mechanisms that may potentially play a role in the resistance. In this study, the susceptibility of *Ae. aegypti* adults to different classes of insecticides used in Singapore was assessed with bioassay, synergism, and biochemical studies. The insecticide susceptibility of mosquito populations from historical and new dengue sensitive areas was compared. Historically sensitive areas were locations where dengue clusters had been present for at least five years when larvae were collected; in these areas, insecticides were frequently used to manage dengue outbreaks. New sensitive areas are those where dengue clusters were reported less than five years before the commencement of larvae collection in 2010. We tested the hypothesis that the resistance level in historical sensitive areas is higher than that in new sensitive areas due to longer period of insecticide exposure in the former.

## Methods

### Experimental design

The Bora-Bora strain of *Ae. aegypti* was used in a baseline assay to define the diagnostic dose of each insecticide. Using the diagnostic dose, which is defined as two fold of lethal concentration that kills 99% of the reference population tested (LC_99_ × 2), the mortality rate, 50% and 99% knockdown time of mosquitoes (KT_50_ and KT_99_) for each field strain per insecticide were determined. In separate experiments, synergists were included to determine their effects on mortality rate at the diagnostic dose of the insecticides. Biochemical assays were performed on the same batch of mosquitoes (F1 generation).

### Mosquitoes

**Figure 1:**
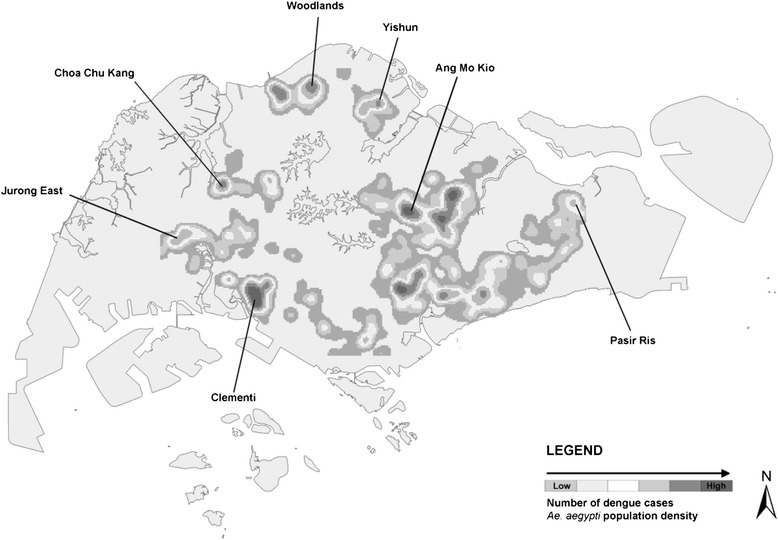
**Map of Singapore showing the sites where populations of** ***Ae. aegypti*** **were collected, the distribution of dengue cases and** ***Ae. aegypti*** **breeding from 2001 to 2007.** Ang Mo Kio, Jurong East and Yishun are historically sensitive areas, whereas the remaining locations are new sensitive areas.

Seven populations of *Ae. aegypti* were collected from public residential areas (i.e. government built residential buildings) in Singapore, from January 2010 to March 2011. These areas were selected based on the relatively high number of reported dengue cases and *Ae. aegypti* indoor breeding sites. The areas were divided into two categories: historical dengue sensitive areas (Ang Mo Kio, Jurong East, and Yishun) where dengue clusters have been reported for more than five years when samples were collected; and new dengue sensitive areas (Choa Chu Kang, Clementi, Pasir Ris, and Woodlands) where dengue clusters were only more recently reported (in less than five years) (Figure 1 and Table 1).

**Table 1:** **Mosquito collection sites in Singapore (2010 – 2011)**

**Locations**	**Collection sites**	**Number of residential buildings #**	**Coordinates**	**Collection period**
Ang Mo Kio*	Ang Mo Kio St 41	5	1°21’N 103°51’E	Jan – Feb 10
Jurong East*	Jurong East St 13	5	1°20’N 103°44’E	Feb – May 10
Yishun	Yishun St 71	4	1°25’N 103°49’E	Jan – Apr 10
Choa Chu Kang	Choa Chu Kang Ave 3	3	1°22’N 103°44’E	Feb – May 10
Clementi	Clementi Ave 3	6	1°18’N 103°45’E	Jan – Feb 10
Pasir Ris	Pasir Ris St 21	4	1°22’N 103°57’E	Jan – Apr 10
	Pasir Ris Dr 6	5	1°22’N 103°57’E	Jan – Apr 10
Woodlands	Woodlands Circle	7	1°26’N 103°47’E	May – Jun 10

*Ave*: Avenue, Dr: Drive, St: Street, *conducted a second collection during the first quarter of 2011. # Ovitraps were set along the corridors at all residential buildings to get the sufficient amount of eggs. Ang Mo Kio, Jurong East and Yishun are historically sensitive areas; others are new sensitive areas.

Eggs from each site were collected and maintained separately as a single colony. To collect the eggs, ovitraps filled with hay-infusion [13] were placed along corridors in shaded areas or near potted plants. The traps were replaced weekly and paddles were air-dried for 1 or 2 weeks prior to hatching of eggs. All emerged adults were identified to species based on morphological characteristics [14], and those identified as *Ae. aegypti* were maintained at 25 ± 2°C, 75 ± 5% relative humidity, and a 10 h light:14 h dark photoperiod with a 10% sucrose solution. Female mosquitoes aged 5–7 d were allowed to blood-feed on live guinea pigs. The use of live animals for laboratory work was approved by the Institutional Animal Care and Use Committee at the Environmental Health Institute, National Environment Agency, Singapore. Biochemical assays were performed using adults, which were killed at −20°C. Bioassay studies were conducted using F2 progenies. The Bora-Bora strain, which is a reference susceptible strain (F112) that has never been exposed to insecticide, was used for comparison. Standard protocols were followed strictly to ensure the production of uniform sized adults.

### Insecticides

Technical grade insecticides (>90% purity) were used in this study. The following insecticides were tested: type I pyrethroid (permethrin (97.1%) from Bayer Cropscience, Bangkok, Thailand); type II pyrethroids (cypermethrin (93%) and deltamethrin (98%) from Asiatic Agricultural Industries, Singapore); non-ester pyrethroid (etofenprox (95.3%) from Jiangyin Trust, Jiangyin, Jiangsu, China), and organophosphate (pirimiphos-methyl (91.5%) from Syngenta Crop Protection, Singapore). To estimate LC_50_ and LC_99_ of each insecticide for each strain, five different concentrations of each insecticide were prepared according to the WHO protocol [15]. Ethanol was used as the solvent.

### WHO bioassays

The tests consisted of two parts: baseline test and diagnostic test. The baseline test was first performed using the Bora-Bora strain to determine the local diagnostic dosages (LC_99_ × 2) to use for the tests of field strains. Susceptibility of the Bora-Bora strain to all insecticides was determined by exposing 5–7 d old non-blood-fed female mosquitoes to varying concentrations of insecticides prepared in ethanol. A batch of 20–25 mosquitoes was aspirated into a plastic holding tube (12 × 4 cm) lined internally with an untreated filter paper (Whatman 12 × 15 cm, evenly impregnated with 3 ml of 10% ethanol and left to air dry for 30 min before being sealed and kept for 24 h prior to the test) and observed for viability for 30 min. Weak and damaged mosquitoes were removed and replaced. Mosquitoes from the holding tube were transferred to a horizontal testing tube (lined internally with a treated filter paper impregnated with 3 ml of insecticide solution) and exposed to insecticide for 1 h. Mosquitoes were transferred back to the holding tube vertically after 1 h of insecticide exposure for recovery, where 10% sucrose solution was provided. Mortality at 24 h post-treatment was recorded. Knockdown was defined as “collapsed against the netting or fallen to the base of the test tube and not moving” [16].

To establish the diagnostic dose (LC_99_ × 2), the Bora-Bora strain was tested using five concentrations of each insecticide in four replicates, to obtain mortality ranges from 0 to 100% to generate LC_50_ and LC_99_ values according to the WHO guidelines [15]. Five controls without insecticide (filter paper treated with 10% ethanol only) were used per test.

To assess the susceptibility of each field population to each insecticide, adults were exposed to the diagnostic dose (obtained from susceptibility baseline) for 1 h and 3 h for organophosphate and all pyrethroid insecticides, respectively. The number of knockdown mosquitoes was observed at 10, 30, 45, 60, 90, 120, 150 and 180 min to obtain the KT_50_ and KT_99_ values. Knockdown time is a more sensitive indicator than mortality rate for resistance detection [17]. Unlike a dose/response test, it requires fewer individuals for toxicity evaluation. Knockdown time (KT_50_), which is the time required for 50% of individuals to be knocked down was used compared to lethal concentration (LC_50_) for resistance when resistance is recessive or present in low frequency [18]. The longer exposure period (3 h instead of 1 h) was used in all pyrethroid bioassays because preliminary tests showed that no knockdown occurred within 1 h. Mosquitoes were held for 24 h in the holding tube after exposure before mortality was recorded. Three tests, each performed on consecutive days, were conducted for each insecticide for each of the seven field populations.

### Synergism tests

Synergism tests were conducted using the field populations to evaluate the effectiveness of synergists on detoxification of insecticides. Piperonyl butoxide (PBO) (94.4%, from Endura Fine Chemicals, Bologna, Italy), S,S,S-tributyl phosphorotrithioate (DEF) (97.1%, from Greyhound Chromatography and Allied Chemicals, Birkenhead, Merseyside, UK), and triphenyl phosphate (TPP) (99%, from Sigma-Aldrich, Singapore) were used. Adult mosquitoes were exposed to each synergist at varying concentrations to determine the maximum sub-lethal concentration. Subsequently, the sub-lethal doses (4%, 5% and 10% for PBO, DEF, and TPP, respectively) were used in synergism tests. The protocol for the synergism tests were similar to the bioassays described above, except that the insecticide was mixed with synergist (1:1) prior to the test. Each synergist was used in conjunction with each of the five insecticides. Diagnostic tests in WHO bioassays section (exposure to insecticide only) served as positive control while bioassays without insecticide (exposure to 10% ethanol only) were used as negative control.

### Biochemical assays

Enzyme levels in individual adults of the same batch of mosquitoes (the Bora-Bora strain, F112, and field strains, F1 generation) were measured according to the WHO procedure previously described by Hemingway [19]. Briefly, non-blood-fed young adult female mosquitoes (<3 d old) were individually homogenized in 200 μl of reverse-osmosis water on ice. Next, 25 μl of homogenate were used in the acetylcholinesterase assay. The remaining homogenate was centrifuged at 14 K, 4°C for 30 s, and the supernatant was used as the enzyme source for all other enzyme assays. A total of 94 adults per strain were analysed. The assays were performed in 96-well microplates on ice, and the absorbance (optical density (OD) values) was measured using a microtitre plate reader (ELISA system, Sunrise™, Tecan®, Mannedorf, Switzerland) with Magellan™ data analysis software (Tecan Group Ltd, Mannedorf, Switzerland). Enzyme activities were calculated as described below.

### Acetylcholinesterase (AchE) assay

For each sample, 25 μl of insect homogenate were mixed with 145 μl of triton phosphate buffer. Next, 10 μl of 0.01 M dithiobis 2-nitrobenzoic acid solution and 25 μl of 0.01 M acetylthiocholine iodide were added to initiate the reaction. Two such reactions were prepared for each sample. While one reaction was allowed to progress, the other was inhibited using 0.05 μl of 0.1 M propoxur. The OD of both reactions was measured at 405 nm after 1 h incubation, and the activity was expressed as percentage insensitive AchE activity after propoxur inhibition [20].

### Non-specific esterase (EST) assay

For each sample, 20 μl of supernatant derived from the insect homogenate were mixed with 200 μl of the substrate, 30 mM α-naphthyl acetate. At the same time, another replicate of the same samples was also incubated with 30 mM β-naphthyl acetate. After 15 mins of incubation at room temperature, 50 μl of fast blue stain were added to each reaction. The OD value was measured at 570 nm 15 min later. The activity against each substrate was calculated from standard curves of absorbance for known concentrations of α-naphthol or β-naphthol. Enzyme activities are expressed as nmole of α-naphthol or β-naphthol/min/mg protein.

### Glutathione S-transferase (GST) assay

A total of 200 μl of 10 mM reduced glutathione (GSH) and 63 mM 1-chloro-2,4-dinitrobenzene mixture was added to 10 μl of supernatant derived from the insect homogenate. Absorbance was determined at 340 nm after 20 min of incubation. GST activity was calculated following Beer’s Law (A = εcl) and is reported as mMole of CDNB/min/mg protein. The OD value (A) was transformed to μmole of CDNB conjugates using the extinction coefficient (ε) of 4.39 mM^−1^. The path length (the depth of the buffer solution in the microplate well, l) was 0.6 cm.

### Monooxygenase (MFO) assay

MFO activity was initiated by the addition of 80 μl of 0.625 M potassium phosphate buffer pH7.2, 200 μl of methanol solution of 3,3,5,5-tetramethyl benzidine solution, and 25 μl of hydrogen peroxide (3%) to 2 μl of supernatant derived from the insect homogenate. The reaction was allowed to oxidise for 2 h at room temperature before the OD value was read at 650 nm. MFO activity was calculated from a standard curve of absorbance for a known concentration of cytochrome C [21]. Enzyme activity is expressed as equivalent units of cytochrome P450/min/mg protein.

### Protein assay

Protein concentration was used as a standard correction factor for the data for all enzyme activities to account for size variances among individuals. The protein concentration was calculated and transformed from the bovine serum albumin standard curve using a commercial kit (Bio-Rad, Foster City, California, USA). For this assay, 10 μl of homogenate were mixed with 300 μl of Bio-Rad dye reagent and incubated for 5 min. The OD was read at 570 nm.

### Data analysis

Two different resistance classifications (mortality percentage and resistance ratio, RR_50_) were used to indicate the susceptibility of *Ae. aegypti* to the insecticides tested in this study. Mortality percentage was used to assess the effectiveness of synergists in enhancing the toxicity of insecticides, whereas the RR_50_ is a more sensitive indicator for resistance detection compared to mortality percentage.

Bioassay results were expressed as mortality percentage and the susceptibility status of each population was classified according to Davidson and Zahar [22], which corresponds to incipient resistance for interpreting the mosquito results on 4% DDT. Insects with 98-100% mortality were classified as susceptible, those with mortality less than 80% were classified as resistant, and those with 80-97% were classified as intermediate. Mortality rates were corrected using Abbott’s formula [23] when control mortality was between 5% and 20%.

The RR_50_ was calculated by dividing the KT_50_ value of field strains with the corresponding KT_50_ value of the susceptible strain. RR_50_ was scaled as follows: RR_50_ < 1 (susceptible), RR_50_ = 1 to 10 (low resistance), RR_50_ = 11 to 30 (moderate resistance), RR_50_ = 31 to 100 (high resistance), and RR_50_ > 100 very high resistance [24].

Mortality percentages and enzyme levels were tested for normality and variance homogeneity using Komolgorov-Smirnov and Levene’s tests, respectively. Non-normal data were arcsine log transformed to stabilize the variance. Two sample *t*-test or Mann–Whitney non-parametric test were applied to test for differences in the mortality between the Bora-Bora strain and respective field strains. Statistical significance was determined at *P* < 0.05. The Mann–Whitney non-parametric test was used to analyse the effect of synergism on mortality. All analyses were conducted using SPSS (PASW Statistics 19) software.

## Results

### Susceptibility status

Table 2 shows the diagnostic doses of each insecticide determined from experiments using the Bora-Bora strain. The toxicity levels of the five insecticides tested decreased in the following order: deltamethrin > cypermethrin > pirimiphos-methyl > permethrin > etofenprox.

**Table 2:** **Diagnostic dose (%) of five adulticides determined using the Bora-Bora strain**

**Adulticides**	**n**	**LC**_**50**_ **(95% CL) (%)**	**LC**_**99**_ **(95% CL) (%)**	**Diagnostic dose (%)**
Cypermethrin	1765	0.017 (0.015 - 0.020)	0.074 (0.056 - 0.108)	0.148
Permethrin	1852	0.067 (0.052 - 0.087)	0.283 (0.165 - 1.638)	0.566
Etofenprox	1861	0.344 (0.236 - 0.499)	3.098 (1.433 - 27.158)	6.196
Deltamethrin	1851	0.002 (0.001 - 0.002)	0.007 (0.005 - 0.015)	0.014
Pirimiphos-methyl	1841	0.033 (0.032 - 0.034)	0.106 (0.095 - 0.123)	0.212

*LC*: Lethal concentrations, *CL*: Confident limits, Diagnostic Dose: LC_99_ × 2.

Table 3 shows the toxicity of the different insecticides against *Ae. aegypti* adults collected from seven locations in Singapore. The RR_50_ values of these field strains to deltamethrin were the highest among the insecticides tested (44.02 to > 142.06-fold higher than control). RR_50_ were moderate to high for permethrin (27.58 to 64.96-fold) and etofenprox (24.75 to 75.64-fold) and low to moderate for cypermethrin (3.76 to 16.94-fold). The lowest was for pirimiphos-methyl (1.01 to 1.51-fold). Among the different combination of mosquito populations and insecticides, the deltamethrin resistance of mosquitoes from Ang Mo Kio and Yishun appeared to be the highest (RR_50_ > 142.06-fold), as no knockdown mosquitoes were observed during the 3 h exposure period. The two areas were historically sensitive areas. The next highest combination involved new dengue sensitive areas, Clementi and Pasir Ris, with populations against deltamethrin, displaying RR_50_ > 130-fold. The rest of the combinations involving the pyrethroids tested ranged from RR_50_ 3.76 to 75.64-fold. Interestingly, there is no significant difference in resistance among populations from historically dengue sensitive areas and new dengue areas (MANOVA test: *F*_4,2_ = 6.489, *P* = 0.138).

**Table 3:** **The toxicity of cypermethrin, permethrin, etofenprox, deltamethrin and pirimiphos-methyl against** ***Ae. aegypti*** **adults collected from seven locations in Singapore**

**Insecticide**	**Strain**	**n**	**KT**_**50**_ **(95% CL) (min)**	**KT**_**99**_ **(95% CL) (min)**	**Slope**	**χ** ^**2**^ **(df)**	**RR**_**50**_
Cypermethrin	Bora-Bora	300	9.99 (9.66 – 10.30)	17.66 (16.64 – 19.03)	9.40 ± 0.60	1.27 (2)	–
	Ang Mo Kio	275	169.31 (138.13 – 238.08 )	1297.44 (661.27 – 5187.65)	2.63 ± 0.15	6.03 (6)	16.94
	Jurong East	297	137.04 (127.08 – 149.20)	1881.81 (1400.88 – 2703.23)	2.04 ± 0.10	1.04 (6)	13.71
	Yishun	249	61.09 (55.94 – 66.32)	381.45 (310.43 – 496.22)	2.92 ± 0.12	1.59 (6)	6.11
	Choa Chu Kang	185	58.80 (51.15 – 66.56)	319.60 (242.12 – 479.45)	3.16 ± 0.15	2.99 (6)	5.88
	Clementi	230	114.64 (108.23 – 121.93)	761.42 (623.20 – 971.87)	2.82 ± 0.14	0.92 (6)	11.47
	Pasir Ris	233	140.9 (113.14 – 201.01)	1099.35 (540.50 – 5522.01)	2.60 ± 0.15	7.79 (6)	14.10
	Woodlands	241	37.58 (21.64 – 52.86)	513.49 (255.84 – 2632.36)	2.04 ± 0.09	13.78 (6)	3.76
Permethrin	Bora-Bora	303	3.84 (3.27 – 4.37)	13.84 (10.96 – 19.96)	4.18 ± 0.18	4.04 (4)	–
	Ang Mo Kio	242	249.77 (193.03 – 375.99)	6551.79 (2650.48 – 30725.79)	1.64 ± 0.12	1.94 (6)	64.96
	Jurong East	241	119.87 (111.36 – 130.04)	1458.47 (1096.47 – 2076.75)	2.14 ± 0.11	1.18 (6)	31.18
	Yishun	247	153.13 (133.12 – 183.35)	2313.47 (1361.99 – 5009.64)	1.97 ± 0.12	1.59 (6)	39.83
	Choa Chu Kang	240	131.55 (122.16 – 142.96)	1395.70 (1055.13 – 1975.95)	2.26 ± 0.12	0.79 (6)	34.21
	Clementi	243	106.06 (91.27 – 126.51)	1251.56 (737.93 – 2900.56)	2.17 ± 0.11	3.21 (6)	27.58
	Pasir Ris	230	204.79 (181.02 – 238.92)	3327.21 (2136.27 – 5968.84)	1.92 ± 0.13	1.42 (6)	53.26
	Woodlands	224	109.01 (95.15 – 127.79)	1110.18 (690.86 – 2308.16)	2.30 ± 0.12	2.65 (6)	28.35
Etofenprox	Bora-Bora	241	7.52 (6.89 – 8.15)	13.76 (11.89 – 17.64)	8.86 ± 0.43	4.36 (4)	–
	Ang Mo Kio	249	568.82 (314.24 – 2713.07)	17186.39 (3294.48 – 1596318.73)	1.57 ± 0.16	3.21 (6)	75.64
	Jurong East	268	506.69 (380.34 – 761.06)	25359.74 (10505.36 – 90910.53)	1.36 ± 0.12	1.08 (6)	67.38
	Yishun	257	221.21 (156.39 – 501.05)	3211.54 (1024.06 – 75816.37)	2.00 ± 0.14	7.55 (6)	29.42
	Choa Chu Kang	248	313.21 (231.34 – 537.29)	5049.65 (1986.78 – 29308.23)	1.93 ± 0.16	2.28 (6)	41.65
	Clementi	246	186.09 (150.86 – 260.53)	2094.00 (1017.04 – 7932.77)	2.21 ± 0.14	3.51 (6)	24.75
	Pasir Ris	247	405.35 (244.78 – 1471.48)	10869.58 (2396.43 – 666897.06)	1.62 ± 0.15	4.21 (6)	53.9
	Woodlands	238	380.72 (252.61 – 896.18)	8489.59 (2476.39 – 128146.80)	1.72 ± 0.16	2.79 (6)	50.63
Deltamethrin	Bora-bora	299	9.51 (8.34 – 10.86)	17.15 (13.77 – 31.96)	9.08 ± 0.49	11.34(4)	–
	Ang Mo Kio	248	NO KD	NO KD			–
	Jurong East	245	651.42 (442.97 – 1287.83)	8016.95 (3151.01 – 42766.59)	2.13 ± 0.31	0.54 (6)	68.48
	Yishun	249	NO KD	NO KD			–
	Choa Chu Kang	250	418.72 (295.23 – 821.19)	4908.35 (1900.67 – 32536.18)	2.17 ± 0.22	1.71 (6)	44.02
	Clementi	249	1351.43 (643.69 – 8844.53)	23809.23 (4765.74 – 1458944.60)	1.86 ± 0.41	0.69 (6)	142.06
	Pasir Ris	237	1301.18 (610.24 – 10278.39)	20376.87 (4004.39 – 1810855.45)	1.94 ± 0.46	0.38 (6)	136.78
	Woodlands		NA	NA			–
Pirimiphos-	Bora-bora	304	107.10 (91.61 – 156.36)	284.96 (182.61 – 880.49)	5.47 ± 0.47	4.71 (8)	–
methyl	Ang Mo Kio	270	128.21 (124.83 – 131.69)	273.99 (255.06 – 298.55)	7.05 ± 0.34	0.79 (6)	1.19
	Jurong East	267	107.99 (100.68 – 115.38)	235.36 (205.10 – 287.59)	6.87 ± 0.29	3.71 (6)	1.01
	Yishun	271	145.10 (140.58 – 150.12)	358.06 (323.54 – 405.62)	5.93 ± 0.32	0.50 (6)	1.35
	Choa Chu Kang	257	156.20 (129.37 – 219.52)	638.08 (366.42 – 2780.33)	3.80 ± 0.21	10.82(6)	1.46
	Clementi	250	161.30 (138.33 – 202.96)	872.08 (531.85 – 2165.77)	3.17 ± 0.18	4.44 (6)	1.51
	Pasir Ris	254	152.90 (145.93 – 161.28)	304.43 (264.53 – 376.96)	7.77 ± 0.48	1.95 (6)	1.43
	Woodlands	246	133.39 (120.85 – 150.10)	519.20 (385.57 – 826.07)	3.94 ± 0.20	3.29 (6)	1.26

*CL*: Confidence limits, *RR*_*50*_: Resistance ratio values are based on KT_50_ levels of the field strain divided by KT_50_ levels of the reference strain (Bora-Bora). Ang Mo Kio, Jurong East and Yishun are historically sensitive areas, whereas the others are from new sensitive areas. *NO KD*: Complete loss of knockdown effect. *NA*: No data were shown due to insufficient number of mosquitoes collected from the location. Chi square (χ^2^) indicates the goodness of fit of the regression line [25].

The mortality rates of field populations, subjected to the diagnostic doses of insecticides, corresponded to the RR_50_ results, with all populations displaying low mortality to pyrethroids and high mortality to pirimiphos-methyl (Table 4). The population most resistant to pyrethroids (<13% mortality) was Ang Mo Kio, a historically sensitive area. The population from this area also had the highest RR_50_ values for all pyrethroids tested (Table 3). Overall, the mortality rates of field mosquitoes exposed to cypermethrin, permethrin and etofenprox were between 1.27% and 68.44%. Although mortality tests were not conducted against deltamethrin due to the limited number of field mosquitoes collected, low mortalities would be expected based on the high RR_50_ values for this insecticide.

**Table 4:** **Susceptibility of** ***Ae. aegypti*** **adults to different insecticides at diagnostic dose (LC**_**99**_ **× 2) and insecticides with synergists from various locations in Singapore**

**Strain**	**Mean % mortality** ^**1**^ **± SE**							
**Insecticide**	**Bora-Bora**	**Ang Mo Kio**	**Jurong East**	**Yishun**	**Choa Chu Kang**	**Clementi**	**Pasir Ris**	**Woodlands**
Total exposed (n)	1261	1239	1331	1175	1126	1184	1198	1208
Cypermethrin only	100 ± 0	1.27 ± 1.27	58.93 ± 8.92	29.50 ± 8.18	34.63 ± 6.03	15.20 ± 2.80	27.53 ± 1.61	68.44 ± 2.55
Cypermethrin + PBO	100 ± 0	1.62 ± 0.36	14.60 ± 5.06*	8.43 ± 2.09	11.60 ± 5.40*	5.41 ± 1.46*	33.83 ± 2.65	32.09 ± 6.15*
Cypermethrin + DEF	100 ± 0	11.03 ± 1.70*	12.67 ± 2.08*	37.19 ± 3.08	27.34 ± 5.77	29.87 ± 3.29*	53.79 ± 4.61*	29.63 ± 9.90*
Cypermethrin + TPP	100 ± 0	0.40 ± 0.40	3.50 ± 0.60*	2.42 ± 0.03*	0.83 ± 0.83*	2.14 ± 0.88*	16.79 ± 7.40	6.13 ± 3.97*
Cypermethrin + 3 synergists		4.54 ± 1.64	8.25 ± 0.94*	13.64 ± 6.30	15.47 ± 6.24	2.93 ± 0.43*	31.51 ± 3.75	35.29 ± 10.56*
Total exposed (n)	1250	1228	1182	1239	1252	1224	1219	1206
Permethrin only	100 ± 0	5.75 ± 0.98	11.59 ± 2.93	16.58 ± 4.22	18.57 ± 3.44	27.39 ± 6.83	6.00 ± 1.32	33.88 ± 7.72
Permethrin + PBO	100 ± 0	3.29 ± 2.21	15.99 ± 6.56	22.45 ± 5.52	14.87 ± 0.61	15.14 ± 1.96	3.94 ± 1.97	7.24 ± 2.51*
Permethrin + DEF	100 ± 0	27.35 ± 5.03*	17.59 ± 3.25	29.21 ± 4.47	16.51 ± 1.97	39.95 ± 14.67	25.61 ± 3.23*	38.47 ± 6.85
Permethrin + TPP	100 ± 0	1.22 ± 0.70*	1.60 ± 1.06*	1.21 ± 0.70*	0.78 ± 0.39*	3.16 ± 1.36*	1.20 ± 0.70*	2.02 ± 0.39*
Permethrin + 3 synergists	100 ± 0	1.62 ± 0.81*	1.21 ± 0.70*	1.19 ± 1.19*	1.16 ± 0.01*	6.43 ± 3.21*	0.42 ± 0.42*	14.77 ± 3.67
Total exposed (n)	1240	1193	1245	1215	1235	1185	1265	1144
Etofenprox only	100 ± 0	12.96 ± 6.68	21.87 ± 18.96	26.04 ± 19.23	16.47 ± 11.66	28.86 ± 6.95	14.40 ± 10.79	14.94 ± 13.05
Etofenprox + PBO	100 ± 0	27.16 ± 8.50	23.90 ± 12.29	47.43 ± 9.19	20.71 ± 3.78	38.67 ± 12.55	22.32 ± 2.21	41.68 ± 11.48
Etofenprox + DEF	100 ± 0	31.58 ± 9.25	27.82 ± 11.22	**55.49 ± 0.53**	41.83 ± 5.03	38.64 ± 9.94	32.35 ± 3.95	56.09 ± 6.46*
Etofenprox + TPP	100 ± 0	7.07 ± 2.28	1.54 ± 1.01	2.08 ± 2.08	2.06 ± 0.43*	3.91 ± 1.04*	4.39 ± 1.42	6.10 ± 3.47
Etofenprox + 3 synergists	100 ± 0	12.15 ± 5.96	19.07 ± 10.92	18.84 ± 1.88	13.62 ± 5.09	15.01 ± 3.93	7.40 ± 1.41	20.71 ± 2.24
Total exposed (n)	1248	1290	1154	1089	1242	1256	1256	1177
Pirimiphos-methyl only	100 ± 0	100 ± 0	100 ± 0	99.25 ± 0.75	100 ± 0	95.98 ± 4.02	100 ± 0	98.41 ± 1.59
Pirimiphos-methyl + PBO	100 ± 0	95.40 ± 4.04	79.82 ± 10.09*	47.55 ± 5.16*	95.78 ± 3.15	88.12 ± 8.37	98.86 ± 1.14	81.79 ± 10.03
Pirimiphos-methyl + DEF	100 ± 0	98.85 ± 0.66	98.80 ± 0.72	93.46 ± 5.95	97.00 ± 1.91	99.58 ± 0.42	99.59 ± 0.41	100 ± 0
Pirimiphos-methyl + TPP	100 ± 0	81.36 ± 7.52*	60.16 ± 11.86*	66.01 ± 10.99*	97.27 ± 1.71	2.27 ± 2.27*	67.96 ± 5.78*	64.92 ± 16.35
Pirimiphos-methyl + 3 synergists	100 ± 0	100 ± 0	55.95 ± 10.72*	2.75 ± 1.41*	2.43 ± 1.90*	50.34 ± 5.44*	8.75 ± 2.43*	28.12 ± 3.94*

^1^Mean% mortality followed by an asterisk symbol indicates significant difference compared to the Bora-Bora strain (p < 0.05, independent t-test). Ang Mo Kio, Jurong East and Yishun are historically sensitive areas; others are new sensitive areas. Number in bold denotes mortality higher than 50%.

### Effectiveness of synergists

No significant increase in mortality rate was detected in most populations after synergist treatment. The exceptions are those from Ang Mo Kio, Clementi, and Pasir Ris, which showed significantly higher mortalities after the addition of DEF to cypermethrin and permethrin, and those from Woodlands, showing a significant four-fold increase in mortality when DEF was added in etofenprox (Table 4). However, despite the statistical significance in the enhancement, the synergist did not recover the toxicity of the pyrethroids, with only 3 data points marginally exceeding 50% mean mortality. DEF treatment to all pyrethroid assays led to a slight, but not significant increase in the mortality of *Ae. aegypti* adults in most strains. Counter-productively, in many populations, the “synergist” antagonised the toxicity of pyrethroids. Most marked is TPP which reduced the mortality rate rendered by all insecticides. Reduction of mortality caused by cypermethrin was as low as 42-fold (Choa Chu Kang population), permethrin as low as 23-fold (Choa Chu Kang population) and etofenprox as low as 14-fold (Jurong East). Treatment with any of the three synergists did not increase the mortality of all *Ae. aegypti* strains to pirimiphos-methyl. In summary, the synergists (PBO, DEF, and TPP) are not effective in enhancing the toxicity of insecticides against local *Ae. aegypti*.

### Biochemical assays

**Figure 2:**
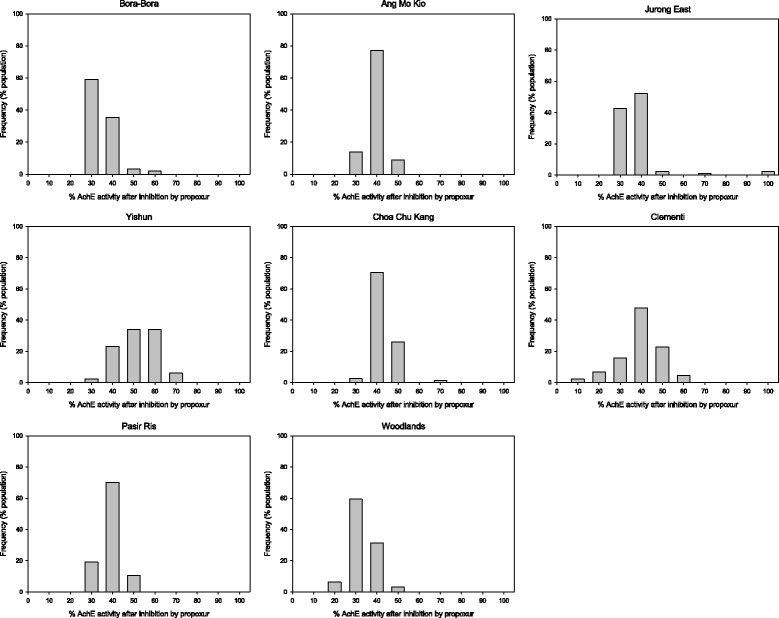
**Altered acethylcholinesterase (AchE) activities in** ***Ae. aegypti*** **adult populations from Singapore.**

On average, adults from all locations except Woodlands showed a very low frequency of altered AchE activity based on the expected 30% propoxur inhibition of AchE activity in susceptible individuals (Table 5) [19]. However, individual *Ae. aegypti* from all locations except Woodlands (*P* = 0.627), exhibited significantly increased altered AchE activity (*P* < 0.05) (Figure 2).

**Table 5:** **Mean (±SE) levels of altered acethylcholinesterase (AchE), non-specific esterases (α- and β-esterase), glutathione S-transferase (GST) and monooxygenase (MFO) activities of** ***Ae. aegypti*** **adults collected from different locations in Singapore**

**Strain**	**n**	**Mean enzyme activities** ^**1**^ **(Mean** ^**2**^ **± SE)**				
		**AchE**	**α-Esterase**	**β-Esterase**	**GST**	**MFO**
Bora-bora	93	30.13 ± 0.67	21.51 ± 0.70	23.61 ± 0.74	0.04 ± 0.00	0.13 ± 0.01
Ang Mo Kio	79	33.98 ± 0.44*	22.23 ± 0.56	23.96 ± 0.81	0.06 ± 0.00	0.02 ± 0.00*
Jurong East	94	32.55 ± 1.08	2.08 ± 1.80*	1.93 ± 1.69*	0.05 ± 0.00	0.53 ± 0.02*
Yishun	82	46.35 ± 0.97*	15.99 ± 0.42*	19.67 ± 0.57*	0.04 ± 0.00	0.22 ± 0.01*
Chua Chu Kang	81	38.31 ± 0.55*	7.85 ± 0.32*	10.60 ± 0.47*	0.05 ± 0.00	0.14 ± 0.00*
Clementi	44	34.25 ± 1.49*	17.78 ± 0.81*	19.09 ± 1.03*	1.99 ± 0.04*	0.13 ± 0.01
Pasir Ris	47	33.85 ± 0.66*	9.54 ± 0.44*	12.67 ± 0.55*	0.04 ± 0.00	0.01 ± 0.00*
Woodlands	32	27.41 ± 1.10*	13.75 ± 0.81*	14.72 ± 0.96*	0.04 ± 0.00	0.20 ± 0.01*

^1^Enzyme activities are expressed as the reaction rate of different substrates/min/mg protein. ^2^Means followed by the asterisk are significantly different compared to the Bora-Bora strain (p < 0.05, two samples t-test or Mann–Whitney test). AchE: percentage insensitive acethylcholinesterase activity after propoxur inhibition. α-esterase: nmole of 1-naphthol/min/mg protein, β-esterase: nmole of 1-naphthol/min./mg protein. GST: mMole of CDNB/min/mg protein. MFO: nmole equivalent unit cyt P450/min/mg protein. The results in bold denotes activity that is more than two times above the control.

**Figure 3:**
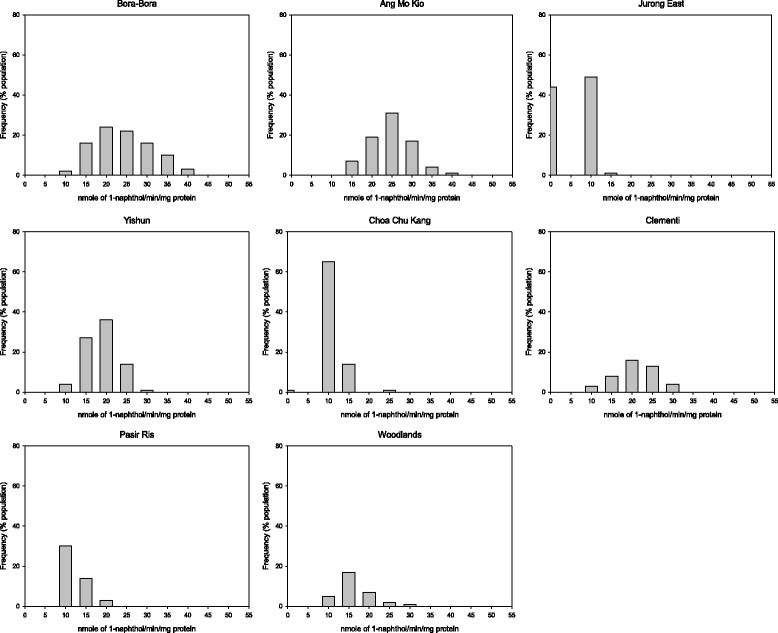
**Esterase activities with α-naphthyl acetate in** ***Ae. aegypti*** **adult populations from Singapore.**

**Figure 4:**
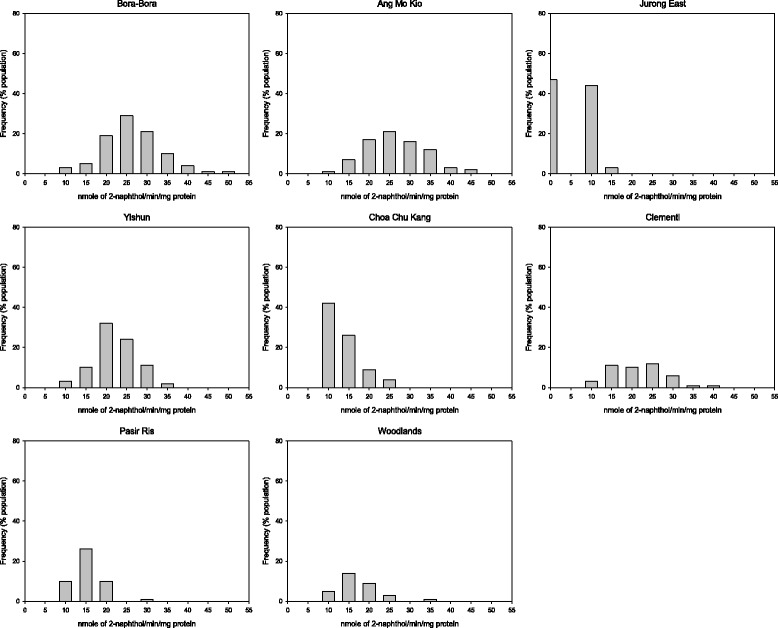
**Esterase activities with β-naphthyl acetate in** ***Ae. aegypti*** **adult populations from Singapore.**

**Figure 5:**
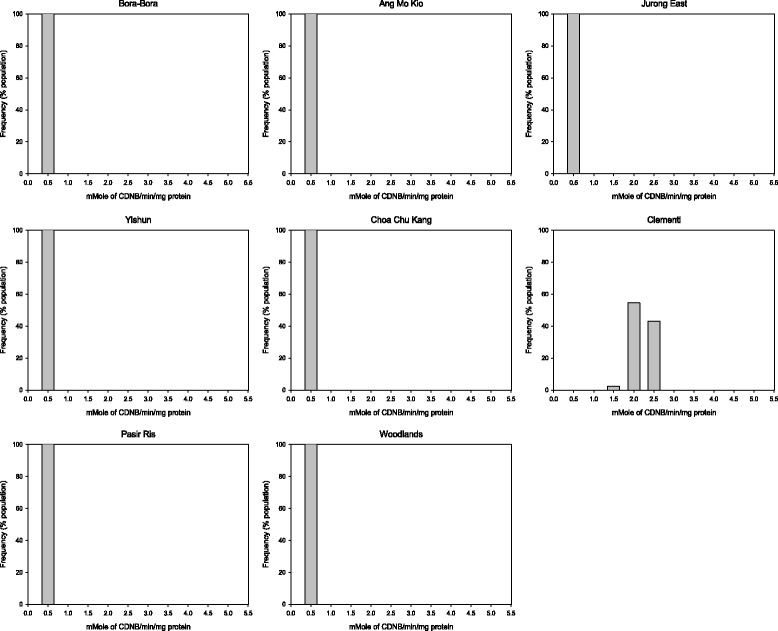
**Glutathione S- transferase (GST) activities in** ***Ae. aegypti*** **adult populations from Singapore.**

**Figure 6:**
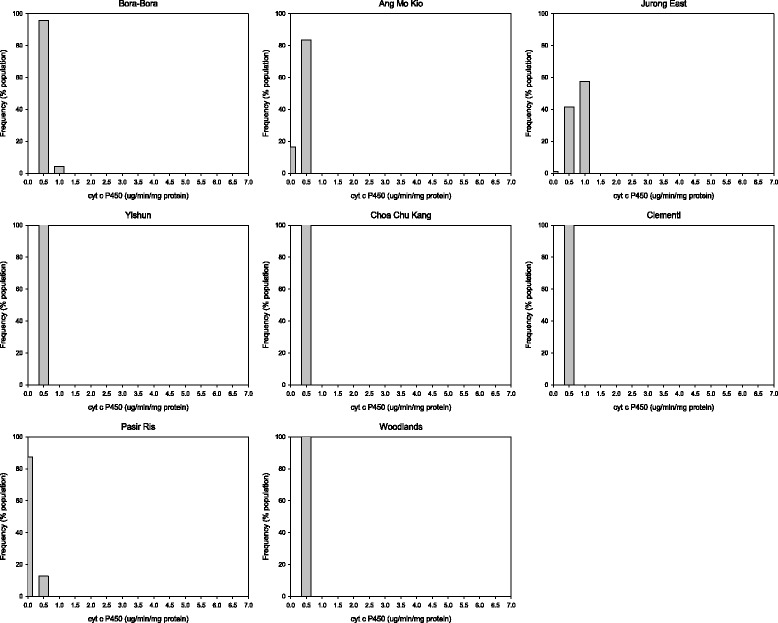
**Monooxygenases (MFO) activities in** ***Ae. aegypti*** **adult populations from Singapore.**

There was no evidence of elevated EST activity in any of the populations tested (Table 5, Figures 3 and 4). The population from Clementi was the only one with elevated GST level (Figure 5). The populations from Yishun, Woodlands and Jurong East exhibited significant increase in mean MFO levels (*P* < 0.05) (Table 5), but the increase in MFO activity was only detected at low frequency at the individual level in Jurong East population (Figure 6).

## Discussion

### Susceptibility status

*Aedes aegypti* populations in Singapore have previously been reported to be resistant to permethrin [26], and a study in 1999 showed that the RR_50_ of field *Ae. aegypti* against permethrin was 12.9-fold of susceptible strain [27]. A study from 2004–2007 has also shown resistance of *Ae. aegypti* to cypermethrin [28]. More recently, we reported the resistance of Singapore *Ae. aegypti* larvae, to a panel of pyrethroids [29]. Detection of resistance among *Ae. aegypti* adult is thus of no surprise. The long term use of pyrethroids for vector control, previous use of DDT which has been found to contribute to today’s widespread pyrethroid resistance and the use of pyrethroids in most household insecticide products, such as aerosols and mosquito coil mats have rendered field selection pressure towards the family of insecticides. This is not unique to Singapore as pyrethroid resistance among *Ae. aegypti* has been reported in many countries [30-34].

However, our study found that *Ae. aegypti* populations from historical and new dengue sensitive areas displayed no significant difference in their susceptibility to all insecticides tested. The results contradicted our hypothesis that the resistance level in historical sensitive areas would be higher than that in new sensitive areas because of more prolonged insecticide exposure in the former. Together with the previous findings of insecticide resistance, these results suggest that the mosquitoes from the newly sensitive areas could be due to migration of the vector from the historically sensitive areas. It is consistent with our observation that emergence of new dengue areas in Singapore is due to the geographical expansion of *Ae. aegypti* on the island. Dispersal of already resistant mosquitoes due to human movement or goods movement could have contributed to the widespread pyrethroid resistance in *Ae. aegypti* throughout the country. This is in contrast to findings from Thailand and Africa [35,36] where insecticide resistance appears to be focal and heterogeneous at short distances in different regions.

Pyrethroids are broadly categorized into three groups based on their structure and toxicology: type I, type II and non-ester pyrethroid. Type II pyrethroids, which contain a α-cyano group, are more toxic than type I pyrethroids [37,38], and our study showed that type II pyrethroids (cypermethrin and deltamethrin) had higher insecticidal activity than the type I pyrethroid (permethrin) (Table 2). Cross resistance among these pyrethroids, due to shared mode of action [39,40] is well known. *Aedes aegypti* in Bandung, Indonesia displayed cross resistance between permethrin (type I pyrethroid), and deltamethrin (type II) and was postulated to be due to these two types of pyrethroids sharing similar chemical structure [41]. In Singapore, resistance to etofenprox (a non-ester pyrethroid) was observed even though it has not been widely used. For example, it represents just 0.17% of insecticides used in fogging by the private pest control industry during the period Jan 2009 to Sept 2011, contrasting with 56.9% due to cypermethrin during the same period. Similarly, Kasai *et al.* [42] reported that the high resistance of *Culex pipiens* to etofenprox was due to cross resistance from permethrin and phenothrin, as etofenprox is rarely used in Japan. *Helicoverpa armigera* also exhibited cross resistance, as it showed different levels of resistance to insecticides to which it had never been exposed [43]. The cross resistance to insecticides with different chemical structure, such as in the case of non-ester pyrethroid (etofenprox) and type II pyrethroids (cypermethrin and deltamethrin), suggest that these pyrethroids may target similar binding sites [37].

In contrast to the high level of pyrethroid resistance detected, all populations of *Ae. aegypti* showed RR_50_ 1.01 to 1.51-fold of resistance to pirimiphos-methyl (Table 3), and this is similar to the 1998 study where the RR_50_ to pirimiphos-methyl was only 1.5-fold [27]. This suggests that the development of organophosphate resistance has not worsened. Rotation of insecticide classes with different modes of action has often been proposed as a strategy to help reduce the selection pressure on the insecticide [44]. However, the cross resistance among different pyrethroids and the widespread resistance revealed by this study suggests that an insecticide rotational strategy would not be a feasible resistance management approach in Singapore.

### Mechanism of resistance

The ineffectiveness of the synergists in alleviating pyrethroid resistance among the Singapore *Ae. aegypti* population indicates the insignificant role of MFOs or other detoxifying enzymes, in the resistance landscape of local *Ae. aegypti*. Several previous studies have suggested that PBO acts as an inhibitor of MFOs [45,46]. However, in Singapore, although DEF increased the mortality rates of four populations rendered by cypermethrin, permethrin or etofenprox, only three data points marginally exceeded 50% mortality rate. This suggests that mechanisms other than the overproduction of detoxifying enzymes, are responsible for the high pyrethroid resistance displayed by local *Ae. aegypti*. The hypothesis is further supported by results from the biochemical assays, where only two populations had any detoxifying enzyme increased more than two times above the control population. It is interesting that most of the populations had enzyme levels below that of the Bora-Bora strain. It has been suggested that greater effect of PBO in susceptible strains may occur because of greater monooxygenases metabolism and higher susceptibility of monooxygenases detoxification in susceptible strain [47].

Nonetheless, the partial synergistic effects of DEF in most populations suggest at least a minor role of EST and GST. DEF is an inhibitor of EST and GST [48]. The synergistic effect of DEF on Clementi population corresponds to the higher GST activity of Clementi population (Table 5, Figure 5). However, the lack of such corresponding results in other populations that also displayed synergistic effect of DEF, suggests the possible involvement of other unknown enzymes that could work in synergy with DEF.

Elevated GST level is known to be associated with DDT resistance [49]. The long-term use of DDT through indoor residual spraying in Orissa State in India resulted in a high GST level in DDT-resistant *Anopheles culicifacies* and *An. annularis* [50]*.* GSTs often act as a secondary resistance mechanism in conjunction with P450- or EST-based resistance mechanisms. *Aedes aegypti* in Singapore is expected to have maintained its high resistance to DDT even though use of DDT was banned in 1973. Ong *et al.* [51] reported that this mosquito species was resistant to DDT in 1980. The elevated GST level in the Clementi population is indicative of DDT resistance in this population. To confirm that DDT resistance is still prevalent among the other field populations, we exposed some field strains to 4% DDT (WHO diagnostic dose). Low mortalities (10.06 - 29.12%, data not shown) were observed. This opens up the possibility that the pyrethroid resistance we encounter today could be a result of cross resistance with DDT. However, as discussed above, the role of GST is limited as demonstrated by non-elevation of the enzyme in most population. The other known cross-resistance mechanism involves the *kdr* mechanism [52-54]. Taken together with the ineffectiveness of synergies, the role of *kdr* in insecticide resistance in local *Ae. aegypti* is suspected, and will be studied.

The antagonistic effect of “synergists” on the toxicity of pyrethroids to some local populations of *Ae. aegypti* is puzzling. Most marked is TPP, which antagonised action of all pyrethroids tested against all populations. Such antagonistic effect has been reported in other studies [55,56]. Martin *et al*. [57] also reported antagonism of toxicity after TPP pre-treatment in *Heliothis virescens*, the tobacco budworm. Pridgeon *et al.* [47] suggested that the increased deltamethrin resistance observed might be due to PBO reducing cuticular penetration of deltamethrin. Alves *et al.* [58] also reported on DEF reducing the toxicity of indoxacarb to *Ostrinia nubilalis*, the European corn borer. There is a dearth of knowledge on the antagonistic effect of chemicals, and more studies are required to shed light on the mechanism of synergism and antagonism. Nevertheless, our results demonstrated the importance of local evaluation of insecticides and synergists, as an inappropriate use of synergist could exacerbate the poor performance due to resistance.

Altered AchE activity is known to confer organophosphate and carbamate resistance in mosquitoes [59,60]. However, the low frequency of altered AchE activity observed in our study indicates that this mechanism is not involved. This supports the bioassay result where low level of pirimiphos-methyl resistance was detected in all locations (Table 3). Elevated levels of EST were correlated with organophosphates and in some cases, pyrethroids. This suggests that *Ae. aegypti* in Singapore are still susceptible to organophosphates although low levels of pirimiphos-methyl resistance were shown.

Our results suggest that multiple mechanisms may be responsible for pyrethroid resistance in *Ae. aegypti*. Despite the resistance displayed in laboratory assays, several *ad hoc* field tests have demonstrated the effectiveness of some of these pyrethroids. While control failure has not been demonstrated in the field, an insecticide resistance management plan must be developed, and insecticides must be used judiciously.

## Conclusions

Insecticide resistance is often a complex dynamic interplay of several mechanisms. Laboratory investigation demonstrated that pyrethroid resistance has developed among *Ae. aegypti* populations in Singapore, though there is no evidence of control failure when these insecticides are used. Susceptibility to pirimiphos-methyl is maintained, but the widespread resistance revealed by this study suggests that an insecticide rotational strategy may not be a feasible resistance management approach in Singapore. Source reduction via environmental management must remain as the mainstay of Singapore vector control programme. Use of insecticides should be judicious, particularly reserving it for control of vector borne diseases.
